# Effect of a home‐based physical activity program on metabolic syndrome in Ghanaian adults with type 2 diabetes: Protocol for a feasibility randomized controlled trial

**DOI:** 10.1002/nop2.2180

**Published:** 2024-05-23

**Authors:** Mohammed Amin, Debra Kerr, Yacoba Atiase, Misbah Muhammad Samir, Andrea Driscoll

**Affiliations:** ^1^ Centre for Quality and Patient Safety, Institute for Health Transformation, Faculty of Health, School of Nursing and Midwifery Deakin University Burwood Victoria Australia; ^2^ Centre for Quality and Patient Safety, Institute for Health Transformation, Faculty of Health, School of Nursing and Midwifery Deakin University Geelong Victoria Australia; ^3^ University of Ghana School of Medicine and Dentistry, National Diabetes Management and Research Centre Korle‐Bu Teaching Hospital Accra Ghana; ^4^ Department of Physiotherapy Korle‐Bu Teaching Hospital Accra Ghana

**Keywords:** exercise, metabolic syndrome, physical activity, randomized controlled trial, type 2 diabetes

## Abstract

**Aim:**

To describe the protocol of a feasibility trial designed to test the preliminary effect of a 12‐week culturally appropriate physical activity programme on metabolic syndrome markers and quality of life in Ghanaian adults with type 2 diabetes (T2DM).

**Design:**

Feasibility randomised controlled trial.

**Methods:**

Through random allocation, 90 adults with T2DM will be allocated to either the control group (CG) (*n* = 45) or the intervention group (IG) (*n* = 45). The IG will receive the physical activity programme in addition to their usual diabetes care; those in the CG will receive their usual diabetes care. Measurements will be performed at baseline and 12‐week follow‐up. The primary outcome is a change in metabolic syndrome markers in the IG compared to the CG. Secondary outcomes are: (a) a change in quality of life in the IG compared to the CG, (b) the feasibility of implementation.

**Results:**

Findings will inform the design of a future large‐scale trial.

**Patient or Public Contribution:**

Patients with T2DM and their healthcare professionals contributed to this study protocol by participating in semi‐structured interviews towards the design of the physical activity programme.

**Clinical Trial Registration Number:**

The trial is registered in the Australian and New Zealand Clinical Trial Registry (registration number: ACTRN12622000323729p).

## 1 INTRODUCTION

Type 2 diabetes mellitus (T2DM) is a metabolic disorder characterised by the inability of the body to use insulin properly (Aschner, 2017). About 79% of adults with diabetes live in low‐resource countries, and the number of adults with T2DM is projected to increase faster in those countries (69% increase) compared to developed countries (20% increase) (International Diabetes Federation, 2019; NCD‐Risk‐Factor‐Collaboration, 2016). Similarly, 80% of deaths associated with diabetes occur in low‐ and middle‐income countries (International Diabetes Federation, 2019).

Over the past few decades, there has been a significant rise in both T2DM prevalence and T2DM‐related deaths in Ghana (Sarfo‐Kantanka et al., 2019). T2DM is one of the leading causes of morbidity and mortality in Ghana (Ghana Health Service, 2018), and a significant number of T2DM cases are often diagnosed at a later stage when disease complications are evident (Danquah et al., 2018). A study by Agyemang‐Yeboah et al. (2019), conducted at the Komfo Anokye Teaching Hospital in Ghana, found approximately 90% of individuals with T2DM in that hospital had metabolic syndrome (MetS); a constellation of cardiovascular risk factors that increases an individual's risk for cardiovascular disease (CVD), stroke and kidney failure (Chuang et al., 2019).

The use of medication alone in the management of T2DM has rarely been successful; hence, healthy lifestyle behaviours are encouraged (Davies et al., 2018). Despite the health benefits of physical activity (PA), most people with T2DM, especially those in low‐ and middle‐income countries, do not meet the recommended daily exercise of at least 120 min of moderate intensity or 75 min of vigorous intensity per week of PA (Oyewole et al., 2014). PA participation is enhanced when programmes are designed with consideration of socio‐cultural norms (Chaabane et al., 2021).

There is lack of evidence on effective PA programmes for people with T2DM in the African context, especially Ghana. Currently, there is no exercise programme in Ghana available for people with T2DM. This underscores the need for the development and testing of a PA programme to improve MetS markers in people with T2DM. The culturally appropriate home‐based PA programme to be evaluated in the current study is informed by the findings of two of our previous studies (Amin et al., 2023a, 2023b). Testing such programme before full implementation in a larger population provides evidence about its efficacy, possible participation rate and safety.

This paper describes the protocol for a feasibility randomised controlled trial (RCT) designed to test the preliminary effect of a 12‐week culturally appropriate home‐based PA programme on metabolic syndrome (MetS) markers and quality of life (QoL) in Ghanaian adults with T2DM. A secondary objective is to evaluate the feasibility of the PA programme. Thus, the findings from this study will provide preliminary evidence to inform the design of a larger multicentre RCT leading to the adoption of a feasible and effective PA programme for Ghanaian adults with T2DM.

## 2 METHODS

### 2.1 Trial design

This study is a single‐site parallel feasibility RCT in which participants will be randomly assigned to either an intervention group (IG) or control group (CG).

### 2.2 Setting

The study will be conducted at the National Diabetes Management and Research Centre (NDMRC), Korle‐Bu Teaching Hospital in Ghana, a country with a national diabetes prevalence rate of 5.0% (International Diabetes Federation, 2019). The number of patients who visit the NDMRC annually is approximately 23,269 (Korle‐Bu‐Teaching‐Hospital, 2016). The outpatient clinic is open Monday to Friday excluding national holidays, with approximately 70 patients per clinic day (personal communication, Dr Atiase, September 2020).

### 2.3 Study population

The sample will include 90 Ghanaian sedentary adults with T2DM and MetS seeking care at the NDMRC. As the sample will be Ghanaian adults with T2DM and MetS, purposive sampling will be used for participant recruitment. For this study, T2DM diagnosis will be confirmed by the participant's physician or nurse at the NDMRC. MetS will be determined according to the definition of the National Cholesterol Education Programme (NCEP) Adult Treatment Panel (ATP) III (Adult‐Treatment‐Panel‐III, 2002). This definition has previously been used in a study conducted in Ghana (Agyemang‐Yeboah et al., 2019).

### 2.4 Eligibility criteria

For participants to be included in the study, they must: (a) have a diagnosis of T2DM; (b) have MetS; (c) receive medical clearance provided by their physician to participate in the exercise programme; (d) have resided in Ghana for at least 2 years; (e) be at least 18 years of age and (f) be sedentary (not achieving 600 metabolic equivalent of task – minutes/week), based on the International Physical Activity Questionnaire – short form (IPAQ‐SF) assessment (Craig et al., 2003).

The following are the exclusion criteria: (a) current medical conditions that preclude physical activity (e.g., significant cardiac/respiratory diseases, severe osteoarthritis or arthritis, angina episodes during rest or with minimal exertion, significant vision loss and limb amputation); (b) frailty index score >3; (c) inability to travel to the NDMRC for supervised training; (d) no access to a mobile phone; or (e) pregnancy.

### 2.5 Study enrolment, recruitment, and screening procedure

An enrolment target of 90 individuals with T2DM will be sought over a 3‐month period, with a target of 30 participants per month. Two recruitment approaches will be used: (a) nurses and consulting physicians at the NDMRC will identify potential participants who visit the NDMRC for their routine diabetes management, and (b) a study flyer will be displayed at the NDMRC.

At the pre‐screening stage, if a potential participant sees the flyer and is interested in the study, they will contact the researcher via the contact details on the flyer, or if they are at the NDMRC, they will speak to a nurse or physician about their interest in taking part in the study. The potential participant will be referred to a research assistant who is independent of the client's care team. If the research assistant identifies the person to be suitable for the study, they will introduce the study to that potential participant and provide them with a Participant Information and Consent Form (PICF). For participants who cannot read, the research assistant will read the PICF in the local language. The research assistant will answer any question that participants may have about the study.

Participants will be encouraged to discuss their involvement in the study with friends, family and other doctors. The research assistant will emphasise the voluntary nature of the study and how their inability to participate will in no way affect the services they receive at the NDMRC. They will also emphasise that participants have the right to discontinue participation in the study at any time before the data has been analysed.

Physicians at the NDMRC will determine whether individuals are able to participate in an exercise programme, as designed for this study. They will review the patient's medical record and provide a medical clearance note on their medical record.

#### 2.5.1 Screening visit

Individuals who are identified as eligible to participate in the study and who express interest in taking part in the study will be invited to a screening visit. At the visit, the research assistant will complete the eligibility screening checklist. If a participant does not meet all the eligibility criteria, they will be ineligible to enrol in the study. Participants who meet the eligibility criteria will be invited to a baseline visit, where additional baseline testing and randomisation will be done.

### 2.6 Informed consent

Prior to laboratory testing, history taking and physical examination, eligible participants will be required to provide their written consent. The research assistant will obtain written consent during the screening visit at the NDMRC. Participants will provide their consent to participate in the study by signing a written consent form.

### 2.7 Randomisation

Through random allocation, 90 participants will be allocated to the CG (*n* = 45) or IG (*n* = 45). A computer‐generated randomisation sequence using EXCEL will be performed with a 1:1 allocation: an equal number of participants in the IG and CG groups. This randomisation will be done by the lead researcher, who has no contact with the hospital or participants. The randomisation sequence will be uploaded into REDCap. After the participant provides their consent, a nurse who is independent of the recruitment process will access the randomisation group from REDCap and advise the participant of their allocated group, either CG or IG.

Due to the nature of the intervention, participant blinding cannot be achieved. However, outcome assessors will be blinded to the group. Different assessors will perform the baseline and 12‐week follow‐up assessments to further reduce the risk of bias.

### 2.8 Interventions

#### 2.8.1 The intervention group

Participants allocated to the IG will engage in a 12‐week PA programme comprising aerobic exercise and resistance training, in addition to their usual diabetes care. The exercise goal will be at least 120 min/week of exercise distributed over at least 3 days/week. In total, participants in the IG will be asked to engage in 36 sessions of exercise over the 12‐week period, with each session lasting between 35 and 45 min. Of the 36 exercise sessions, 14 individualised sessions will be conducted at the physiotherapy department of the Korle‐Bu Teaching Hospital. The remaining 22 exercise sessions will be self‐delivered at home. Each exercise session will be structured as follows:

1. 3 min of warm‐up (stretching exercises);
2. 10 min of resistance exercise (exercises targeting upper and lower large body muscles using TheraBand). The specific resistance exercise prescribed in this program will be: chest press, shoulder curls targeting the upper body muscles, and leg press targeting lower body muscles;
3. 20–30 min of aerobic exercise (walking) at moderate pace (4.5–5.1 km/h = 3.5 MET) or brisk pace (5.2–5.6 km/h = 4.3 MET) (Ainsworth et al., 2011); and
4. 2 min of cool‐down (stretching exercises).

##### Baseline visit and exercise demonstration

The programme comprises a 45‐min education session that will include content relating to the importance of PA, including recommended PA targets, and the PA intervention (e.g., type, intensity, duration and frequency). The education session will be organised during the participants' baseline visit to the NDMRC and will be led by a nurse and a physical therapist (PT). The education session will teach participants about self‐regulation in relation to the PA programme. Self‐regulation, as explained by social cognitive theory, relates to the ability of the individual to set realistic goals to achieve certain behaviours (Bandura, 1989). Participants will also be advised about prevention of injury (safety issues), overcoming challenges, and sources of social support.

##### Week‐1

In Week‐1, all three training sessions will be done at the physiotherapy department of the Korle‐Bu Teaching Hospital under the supervision of a PT. The reason for the supervised exercise session is to ensure that participants can perform each type of exercise correctly and within the recommended safety standards. At the start of week 1, participants will be assessed to confirm that they are well enough to participate. Each participant will be assessed by the PT for the following measures: (a) FBG, (b) BP and (c) HR. Target HR will be based on the recommended safe range of 64%–76% of maximum heart rate, which is determined by subtracting the participant's age from 220 (Physical Activity Guidelines Advisory Committee, 2009; Riebe et al., 2018). Exercise in week 1 will be limited to 35 min (20 min of walking and 10 min of resistance training plus 5 min of warm‐up and cool‐down) targeting 50%–60% of maximum heart rate.

To prevent the incidence of hypoglycaemia, participants will be advised, during the education session, on the common symptoms of hypoglycaemia, such as abnormal sweating, shakiness, visual disturbance weakness, headache and confusion. Where a participant experiences such symptoms, the participant is instructed to stop the training immediately, take a rest and eat immediately. To prevent hypoglycaemia during exercise sessions at home, participants were also advised on recommended strategies such as not exercising beyond the recommended intensity and time limits (Fahey et al., 2012), performing resistance exercise before aerobics (Colberg et al., 2016) and injecting insulin at least 60 min prior to exercise (for those using insulin) (Koivisto & Felig, 1978). During the weekly exercise sessions at the physiotherapy department, the PT utilised the glucose management strategy recommended by Riddell et al. (2017). Guided by the strategy, if a participant's starting glycaemia is less than 90 mg/dL, they will be instructed to take 10–20 g glucose and delay exercise until glycaemia is greater than 90 mg/dL. If a participant's glucose is between 90 and 124 mg/dL, they will be instructed to take 10 g of glucose. Participants were not allowed to exercise if their starting glycemia was above 270 mg/dL.

##### Week 2 to week 12

Exercise will be adjusted to 45 min (30 min of walking and 10 min of resistance training plus 5 min of warm‐up and cool‐down) at 75% of maximum heart rate. Participants will continue to engage in three exercise sessions per week, having two sessions self‐administered at home and one supervised session at the physiotherapy department, with no 2 days without exercise.

Resistance training will be graduated from 60% one‐repetition maximum (1RM) and progress to 100% IRM based on the assessment by the PT. The one‐repetition maximum measure refers to how much weight the participant can lift in one repetition. Participants will perform a single set of resistance exercises with 8–12 repetitions, with a between‐set resting interval of 90–120 s.

#### 2.8.2 Control group

The CG will maintain their usual diabetes care, including glucose‐lowering medication, dietary management, footcare and regular outpatient care. They will be encouraged to continue their usual PA but will not be referred to a PA programme. They will not be contacted during the RCT and will attend all medical appointments as organised by hospital staff.

### 2.9 Study outcomes

#### 2.9.1 Primary measures

The primary outcome is a change in MetS markers (BP, TG, WC, FBG and HDL) in Ghanaian adults with T2DM who participate in a 12‐week PA programme, compared to those who receive usual diabetes care.

#### 2.9.2 Secondary measures

Secondary outcomes are: (a) a change in QoL in Ghanaian adults with T2DM who participate in a 12‐week PA programme, compared to those who receive usual diabetes care, (b) feasibility of implementation, determined by measures of safety (c) feasibility of implementation, determined by measures of participation.

### 2.10 Data collection

The following data will be collected.

#### 2.10.1 Demographics

Personal information will be collected including age, education, income, marital status, sex and employment. Data will be collected using the Demographic Questionnaire. This data will be collected by a member of the research team during the baseline visit.

#### 2.10.2 Medical history

Data relating to current medications and medical conditions will be collected during the baseline visit and recorded on the medical clearance report.

#### 2.10.3 Quality of life assessment

Quality of life will be assessed using the Short Form Health Survey, version 2 (SF‐12 V2). This tool has 12 items that measure a person's subjective view of their functional health and general well‐being. It measures the following eight specific health domains, including physical functioning, social functioning and mental health. It is assessed based on a 0–100 score in each domain as well as the physical component summary (PCS‐12) and the mental component summary (MCS‐12) (Ware et al., 2002). A higher score indicates high well‐being and vice versa. The questionnaire has good reliability and validity, with an established Cronbach's alpha of 0.82 and 0.81 for PCS and MCS, respectively (Lam et al., 2013). QoL data will be collected at baseline and the 12‐week follow‐up visit.

#### 2.10.4 Physical activity level

Participants will be assessed for their level of PA (e.g., amount of time they undertook PA, type of PA) in the last 7 days using IPAQ (Craig et al., 2003). This data will be collected during the screening visit, to determine if they are sedentary (i.e. not achieving at least 600 metabolic equivalent of task – minutes/week based on the IPAQ assessment). PA level will also be measured at the end of week 12. The validity and reliability of the IPAQ—SF were assessed in a study conducted in 12 different countries (Craig et al., 2003). South Africa was the only African country included in that study. The study found a total reliability coefficient correlation (Spearman's rho) of 0.84 per total MET/ week across the PA domains.

#### 2.10.5 Physical, clinical and biochemical data

Blood pressure and heart rate will be taken during the baseline visit for all participants and during each exercise session at the physiotherapy department for participants in the IG group. Blood pressure will also be measured at the 12‐week follow‐up. WC will be measured at baseline and at the 12‐week follow‐up. A blood sample will be collected at baseline and at the 12‐week follow‐up to measure Fasting Blood Glucose (FBG), high‐density lipoprotein (HDL) and triglycerides (TG).

#### 2.10.6 Exercise participation and complications

Data regarding participation in the PA programme will be collected. This includes the type of exercise, duration, intensity and frequency of those exercises. Data regarding exercise‐related complications will also be collected. The PT will collect such data from participants in the IG each week during their visit to the physiotherapy department and during the week‐12 follow‐up visit.

#### 2.10.7 6‐Minute walk test

The PT will take participants through a *6‐Minute Walk Test* (6MWT) to test their exercise capacity at baseline and 12‐week follow‐up. It assesses baseline functional capacity and cardiovascular risk, which are necessary in prescribing exercise intensity for patients (Amaricai, 2020). The walk test supports the prescription of an effective and safe exercise programme for people with T2DM (Nolen‐Doerr et al., 2018). A study among patients with T2DM showed a high interclass correlation coefficient for the 6MWT (Alfonso‐Rosa et al., 2014).

#### 2.10.8 One‐repetition random motion test

Each participant will be assessed for one‐repetition maximum in each of the resistance exercises. When using a TheraBand, 1RM is measured based on band extension from its original length. A 50%, 75% and 100% increase in band length corresponds to 1.5 kg, 1.9 kg and 2.7 kg, respectively (Kwon et al., 2010). This test will be done at baseline.

### 2.11 Data collection procedure

Data relating to demographics, eligibility screening, PA levels and QoL will be collected using online questionnaires. A nurse or research team member who is independent of the participant's care team will provide the participant with a quiet place at the NDMRC and administer the eligibility screening checklist. Similarly, the nurse will follow the same procedure to administer the baseline demographic data questionnaire and the baseline and week‐12 follow‐up IPAQ—SF and SF‐12V2 questionnaires. If a participant is unable to read and/or write in English, the nurse will administer the questionnaires verbally in the local language. Surveys will be completed electronically via a RedCap survey tool.

Prior to each exercise session at the physiotherapy centre, the PT will measure the IG participants' BP, HR and random blood glucose (RBG) using the hospital's standard procedure.

WC will be taken by measuring the mid‐point between the lower costal margin and iliac crest.

A 5‐mL sample of venous blood will be collected after at least an 8‐h overnight fast. Participants will be advised about the need for the overnight fast in the PICF and will be sent a reminder via text. A senior biomedical scientist will collect blood samples and biochemical tests (TG, HDL and FBG) will be performed using the hospital's standard protocol. Blood sample will be collected into a serum separation tube and analysed using a dry chemistry analyser.

During the participants' weekly visit to the physiotherapy department, the PT will assess exercise participation and safety using a checklist for exercise participation and the participants' exercise diary. Measures of participation will be determined by the percentage of sessions attended and the proportion of participants who complete the prescribed PA: 22 sessions of home‐based aerobic exercises and 14 sessions of facility‐based resistance training. An overall score <50% of attendance will be judged as low participation and above 70% will be graded as high participation. A score between 50% and 70% will be graded as moderate participation. Reasons for non‐participation or withdrawal will be documented.

Safety outcomes will be assessed by examining participant's subjective reports relative to possible pain, injury, medical emergencies and other exercise‐related complication that may be encountered during participation in the PA program. Safety and participation rate will be assessed as a composite of any adverse events recorded as part of the PT's checklist for exercise participation and safety and the participants' exercise diary.

**FIGURE 1:**
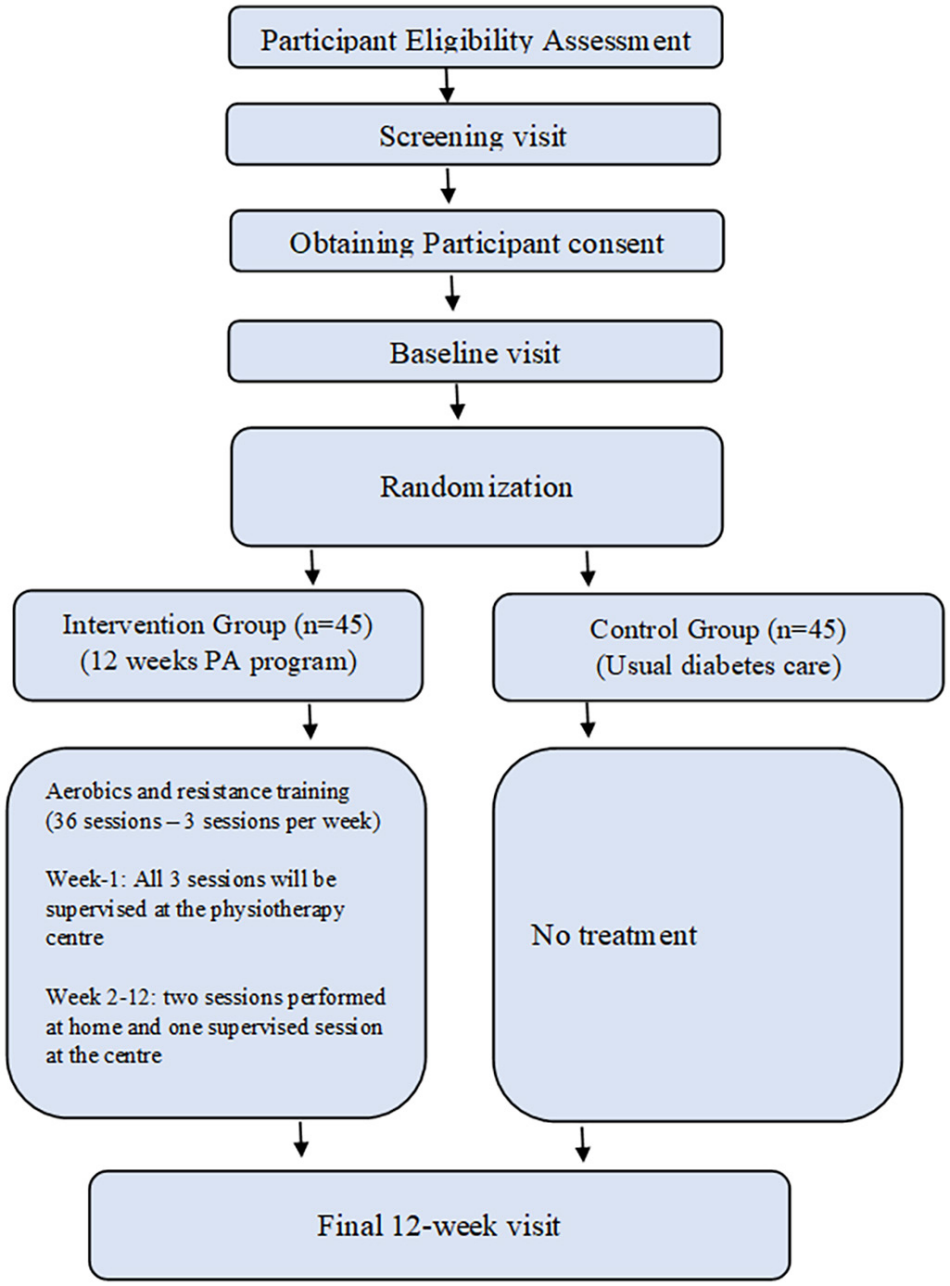
Participant flow diagram.

At the end of each training session, the Borg Rating of Perceived Exertion (RPE) scale will be used to assess perceived exercise tolerance and exercise fatigue (Liguori et al., 2021). Schedule of visits for the RCT are outlined in Figure 1.

### 2.12 Sample size

Feasibility studies are designed to obtain preliminary data (Eldridge et al., 2016). Billingham et al. (2013) examined sample size for feasibility RCTs and found a median sample size of 72 (36 per arm). They concluded that while it is important to justify sample size in feasibility RCTs, calculating sample size may not be appropriate. Thus, this proposed study will aim for a sample of 72 participants. To account for attrition, a 20% adjustment will be added. Therefore, the target sample size for this feasibility study will be 90 participants: 45 in each group.

### 2.13 Data analysis

Data analysis will be performed using the latest version of STATA. Feasibility outcomes will be summarised using descriptive statistics such as frequencies, percentages, mean, SD, median and interquartile range. Relevant parametric and non‐parametric repeated measure analyses will be undertaken to measure between groups differences and changes in effect between and within the two groups. Specifically, the student's *t*‐test will be used for comparing the IG and CG. Between group analysis will be conducted using Pearson's chi‐square and Mann‐Whitney tests for categorical and continuous variables, respectively. A *p*‐value of <0.05 will be considered statistically significant. To obtain the overall effect of the PA intervention on MetS markers, the MetS score for each participant will be assessed. The MetS score is a useful measure of MetS severity, and it correlates to the severity of CVD (Solymoss et al., 2004). The MetS Z‐score will be calculated by adding the MetS risk components for each participant. The MetS Z‐score would be used to measure the change in MetS severity (DeBoer et al., 2018). Further, the effect of the programme on individual MetS markers will also be examined.

### 2.14 Ethical review

Ethics approvals for the study were sought from the following institutions: Institutional Review Board of the Korle‐Bu Teaching Hospital (protocol ID number: KBTH‐IRB/00011/2022) and the Deakin University Human Research Ethics Committee (Project Number 2022‐041). The trial is registered in the Australian New Zealand Clinical Trial Registry (registration number: ACTRN12622000323729p). This study is guided by the Consolidated Standards of Reporting Trials (CONSORT).

## 3 EXPECTED RESULTS

It is expected that this trial will provide preliminary data regarding the feasibility and safety of the PA programme for Ghanaian adults with T2DM. It will also provide data on outcomes that will inform the design of a future large‐scale RCT.

## 4 DISCUSSION

Interventions aimed at controlling MetS markers in people with T2DM are a priority (Longo‐Mbenza et al., 2010). Despite the integral role PA plays in modifying MetS markers, the problem of meeting recommended PA targets remains a challenge for people with T2DM (Oyewole et al., 2014). A significant proportion of Ghanaian adults with T2DM may be considered high‐priority population due to some health beliefs that influence their health‐seeking behaviours, including PA uptake. For instance, Korsah et al. (2022), in assessing the experiences and cultural beliefs of Ghanaians with diabetes, found that some people perceive their diabetes diagnosis as a spiritual disease that can only be treated through spiritual healing. There is also some evidence that many people in Ghana first seek herbal and faith‐based treatment and only resort to biomedical treatment when the disease persists (Arias et al., 2016). These evidences suggest that PA is perceived as less important in managing diabetes. The education component of the PA programme to be evaluated in this study aims at addressing the poor understanding and other barriers that exist in engaging in a PA programme. Also, lack of access to exercise facilities and the cost to access them appear to be major barriers to PA uptake (Higgerson et al., 2018). The PA programme to be evaluated is expected to be suitable for people in low‐resource communities because it is home‐based with no access cost.

A feasibility RCT is usually intended to guide the planning of a future definitive investigation, as it helps to establish an effect and variance (Thabane et al., 2010). This study has the potential to provide information on efficacy, intervention participation and safety (Eldridge et al., 2016; Thabane et al., 2010). Thus, the findings will inform future trials on study design, relevant resources, trial personnel and data management (Thabane et al., 2010). The results of this feasibility study will provide preliminary evidence regarding the effect of a culturally appropriate PA programme and whether it will show a trend towards improvement in MetS markers and QoL in Ghanaian adults with T2DM. The findings of this study will also prove the feasibility or otherwise of the PA programme, prior to its full implementation in the population.

### 4.1 Limitations

A limitation of this study is that it is a single‐site feasibility RCT, which is part of a PhD project with limited resources, time and funding. Another limitation is that there is no sample size calculation. We adopted an a priori, which recommended a sample size of *n* = 72 (36 per arm), thereby limiting the generatability of the findings to other study settings. Further, due to limited funding, objective PA assessment, such as the use of pedometers could not be utilised. Therefore, home‐based exercises will be assessed based on subjective reports from participants.

## 5 CONCLUSION

An effective secondary preventive strategy is needed to reduce the vulnerability of people with T2DM in Ghana. This study represents one of the first attempts to explore an effective PA programme, that improves MetS markers in this population. It is expected that the findings will provide data on outcomes that will inform the design of a future large‐scale RCT leading to the adoption of a feasible and effective PA programme for Ghanaian adults.

## AUTHOR CONTRIBUTIONS

MA contributed to identifying the research problem, designing the study, preparing the study protocol for ethics application and writing the manuscript. DK contributed to designing the study, preparing study protocol for ethics application and providing feedback on the manuscript. YA contributed to the designing the study, preparing the study protocol for the ethics application and providing the feedback on the manuscript. MMS contributed in designing the physical activity programme, preparing study protocol for ethics application and providing feedback on manuscript. AD contributed to designing the study, preparing the study protocol for the ethics application and providing feedback on the manuscript.

## FUNDING INFORMATION

This study is supported by Deakin University.

## CONFLICT OF INTEREST STATEMENT

The authors declare no conflicts of interest.

## Data Availability

Data sharing not applicable to this article as no datasets were generated or analysed during the current study.
